# Potential Role of miR-196a and miR-196b as Prognostic Biomarkers of Survival in Head and Neck Squamous Cell Carcinoma: A Systematic Review, Meta-Analysis and Trial Sequential Analysis

**DOI:** 10.3390/life12081269

**Published:** 2022-08-19

**Authors:** Mario Dioguardi, Stefania Cantore, Diego Sovereto, Lucia La Femina, Giorgia Apollonia Caloro, Francesca Spirito, Salvatore Scacco, Michele Di Cosola, Lorenzo Lo Muzio, Giuseppe Troiano, Andrea Ballini

**Affiliations:** 1 Department of Clinical and Experimental Medicine, University of Foggia, Via Rovelli 50, 71122 Foggia, Italy; 2 Independent Researcher, Sorriso & Benessere-Ricerca e Clinica, 70129 Bari, Italy; 3 Unità Operativa Nefrologia e Dialisi, Presidio Ospedaliero Scorrano, ASL (Azienda Sanitaria Locale) Lecce, Via Giuseppina Delli Ponti, 73020 Scorrano, Italy; 4 Department of Basic Medical Sciences, Neurosciences and Sensory Organs, University of Bari “Aldo Moro”, 70124 Bari, Italy; 5 Department of Precision Medicine, University of Campania “Luigi Vanvitelli”, 80138 Naples, Italy

**Keywords:** head and neck squamous cell carcinoma (HNSCC), non-coding RNA, miR-196a, miR-196b, oral cancer, trial sequential analysis (TSA)

## Abstract

The etiopathogenetic mechanisms involving tumor genesis, including alteration of cell proliferation, apoptosis, invasion, migration, and death, may lead to alterations in microRNAs (miR) expression. The hypothesis is that with the presence in the literature of recent studies conducted on miR-196a and miR-196b, it is possible to clearly determine, by aggregating the results, whether miR-196 upregulation in head and neck squamous cell carcinoma (HNSCC) tissues can represent a prognostic biomarker of survival through hazard ratio (HR) analysis. The systematic review was conducted following the indications of the PRISMA, and four electronic databases were used (Science Direct, SCOPUS, PubMed, and Cochrane Central), with the addition of gray literature. Combinations of keywords were used, such as miR-196, miR-196 AND HNSCC, microRNA AND HNSCC, LSCC AND miR-196, OSCC AND miR-196, OPSCC AND miR-196, HSCC AND miR-196. The meta-analysis and trial sequential analysis (TSA) were performed using RevMan 5.41 software and Stata 13 (StataCorp, College Station, TX, USA) with the implementation of the R 4.2 software. This search identified 1593 reports and, at the end of the selection, five articles were inserted. The results of the meta-analysis report an aggregate HR for overall survival (OS), between the highest and lowest miR-196 expression of 1.67, 95% CI: [1.16, 2.49]. In this meta-analysis, we found that the forest plot is in favor of higher OS in HNSCC patients, compared with the control, with low miR-196 expression, correlating this data with a favorable prognosis, which indicated the potential role of this miRNA in strengthening the therapy sensitiveness of the HNSCC patients. Consequently, the present systematic review places itself, together with other systematic reviews on this topic, in a key role to the finding of Phase 3 clinical trials studies, in search for a prognostic model of miR-196 for HNSCC. In conclusion, with the limitations of the meta-analysis, it can be argued that miRs of the miR-196 family could be independent prognostic biomarkers of survival for HNSCC.

## 1. Introduction

Between the tumors of the head and neck region, the squamous cell variant (HNSCC) is the most common, and represents one of the main neoplasms affecting humans. In fact, it is estimated that HNSCC represents about 6% of the causes of cancer death and is the sixth most common cancer in the world, with a higher incidence in males [1]. HNSCC are neoplasms that, depending on the epithelium of origin, are recognized as laryngeal squamous cell carcinoma (LSCC), oropharyngeal squamous cell carcinoma (OPSCC), hypopharyngeal squamous cell carcinoma (HSCC), and oral squamous cell carcinoma (OSCC) [1].

The main risk factors are represented by smoking [2] and alcohol consumption, with an important correlation for LSCC with the papilloma virus (HPV 16, HPV 18) [2,3].

MicroRNAs (miRNA, miR), are a large group of small single-stranded non-coding endogenous RNAs approximately 18–25 nucleotides in length that play a significant role in the post-transcriptional regulation of genes through the interaction with 3′UTR of target mRNA [4].

Alterations in the expression of miRs can manifest themselves with changes that lead to their upregulation or downregulation. The main upregulated miRs associated with HNSCC, are miR-375, miR-1234, miR-103, miR-638, miR-200b-3p, miR-191-5p, miR-24-3p, miR-572, miR-483-5p, miR-20a, miR-22, miR-29a, miR-29b, mir-let-7c, miR-17, miR-374b-5p, miR-425-5p, miR-122, miR-134, miR-184, miR 191, miR-412, miR-512, miR-8392, miR-21, miR-31, miR-155 miR-196a, and miR-196b, while the following miRs would be downregulated: miR-9, miR-29c, miR-223, miR-187, Let-7a, miR 27, miR 34, miR 92, miR 124, miR 125a, miR 136, miR139 miR 145, miR 146a, miR 200, miR-195, and miR 205 [5,6].

In particular, the over-regulation of miR-21 [7,8], miR-155 [9], and miR-31 [10] would be associated with a worse prognosis, and among the downregulated is reported miR-195 [11]. Confirmation of these data also comes from recent systematic reviews with meta-analysis, conducted on the prognostic value of these groups of microRNAs [12]. In a similar way, more miRs are associated with a possible use as a prognostic biomarker of survival in HNSCC, and among these were recently highlighted the miR-196a and the miR-196b [13].

These two non-coding RNAs belong to the miR-196 family and, in their mature form, they differ for a single nucleotide and are transcribed by three different genes: *miR-196a1* on chromosome 17q21.32; *miR-196a2* on chromosome 12q13.13, the two mature forms of miR-196a are identical (3′GGGUUGUUGUACUUUGAUGGAU-5′); *miR-196b* on chromosome 7p15.2 (3′-GGGGUUGUUGUCCUU-UGAUGGAU-5′) [14].

Both forms are found to be overexpressed in several tumors such as in the esophageal tumor [15], the small cell lung cancer [16], ovarian cancer, gastric cancer [17], OSCC [18], and pancreatic cancer [19]. The over-regulation of miR-196 could favor proliferation, cell invasion, lymph node metastasis, blockage of apoptosis, and resistance to chemotherapy and radiotherapy. In the literature there is no univocity; indeed, the miR-196 would be found downregulated with oncosuppressive function in some experimental studies and for some types of tumors such as melanoma [20], astrocytoma [21], osteosarcoma [22], and myeloma [23].

Among the main target genes of miR-196 we found HOX family, MAMDC2, CDKN1B, ING5, RAD23B, NFKBIA, ANXA1 [24], NTN4, GATA6, IκBα, ZMYND11, SNHG3, CADM1, GAS5, FOXP2, FOXO1, SOCS2, NME4, and RDX [24]. The miRBase database is the primary public repository and online searchable database of published miRNA sequences and annotation [25]. Each entry in the miRBase Sequence database represents a predicted hairpin portion of an miRNA transcript (termed mir in the database), with information on the location and sequence of the mature miRNA sequence (termed miR). Both hairpin and mature sequences are available for searching and browsing, and entries can also be retrieved by name, keyword, references, and annotation. All sequence and annotation data are also available for download. Each database entry is identified by a stable accession number in addition to the miRNA gene name. The Registry will assign a name only after a paper describing the sequence has been accepted for publication [26,27].

The miRBase also acts as a portal for third party information about microRNA genes and sequence, linking out to other resources such as those that include predicted and experimentally validated targets of microRNAs.

The miRBase does not curate or collate predicted or validated target sets, but rather links from entries to external target resources. Worldwide scientists daily work to improve and increase these links from miRBase. Over a fifth of mature microRNAs (10,609/48,860) in miRBase have links to target predictions, and 4154 (8.5%) link out to validated target sets. Those proportions are much higher for the best-studied and most viewed organisms—for example, 2578 and 2599 of the 2654 human mature sequences in miRBase have links to predicted and validated targets, respectively [25].

Specifically, all the target genes for miR-196a and miR-196b are available as Supplemental Files and the data are also available on the website (https://www.mirbase.org/, accessed on 5 August 2022) [25,26,27].

The diagnostic potential was also investigated by a recent study on circulating miR-196a and miR-196b in plasma samples of 90 patients with oral cancer, identifying the area under receiver operating characteristic (ROC) curve (AUC) of 0.963 [28]. These recent studies showed that miR-196 is over-regulated in HNSCC tissue: in fact, Zhao et al. [29] indicated miR-196b as a potential prognostic factor on a court of 113 LSCC patients with an hazard ratio (HR) between high and low expression of 1.673 C.I. (1.098–2.54), *p* = 0.017 (univariate analysis).

Taking into account these premises, the aim of this systematic review and meta-analysis was to investigate the prognostic potential of mIR-196 as a survival biomarker for HNSCC in the light of new miR-196 studies.

## 2. Materials and Methods

### 2.1. Protocol

The drafting of the review was carried out following the indications of the PRISMA (Preferred Reporting Items for Systematic Reviews and Meta-Analysis) [30], the protocol with which the systematic review performed was established before proceeding with the search and screening of records on data banks. The protocol was in fact registered in advance on PROSPERO (International Prospective Register of Systematic Reviews), with registration number CRD42022332782, and the systematic review was conducted according to the recommendations of the Cochrane Handbook [31].

### 2.2. Eligibility Criteria

The search for sources was directed towards all retrospective, prospective, and randomized trials that investigated the role of miR-196 in tumor tissues and HNSCC, and there also had to be a clear correlation with prognostic survival indices including OS (overall survival), DFS (disease-free survival), PFS (progression-free survival), RFS (relapse-free survival), CSS (cancer-specific survival), and the expression of miR-196.

The formulated PICO question was as follows: Is there a difference in prognostic indices of survival between HNSCC patients with high tissue miR-196 expression versus those with low expression? The different points investigated were (P) participants (patients with HNSCC), (I) intervention (altered expression of miR-196 in HNSCC), (C) control (patients with HNSCC who have low expression of miR-196), and (O) outcome (difference in survival prognosis between patients with low and high miR-196 expression in HNSCC).

The exclusion criteria were (1) studies published in a language other than English; (2) all studies that did not report data on tissue miR-196 expression; (3) all studies that did not report prognostic indices of survival; (4) all literature reviews (considered as bibliographic sources only), case reports, and case series.

Therefore, the inclusion criteria of potentially eligible articles were to include those studies which investigated tissue miR-196 (miR-196a, miR-196b) in relation to prognostic survival indices (OS, DFS, PFS, RFS, CSS) for HNSCCs, reporting relative risk (RR), hazard ratio (HR), Cox regression, or Kaplan–Meier survival curves data.

### 2.3. Sources of Information, Research, and Selection

The research of the studies involved 4 independent reviewers (M.D., D.S., S.C., and L.L.F.). The research and selection phase of the articles was carried out in 3 phases: in the first phase, the inclusion and exclusion criteria, the databases, the keywords to be used, and the period of time in which to conduct the search were jointly decided. In the second phase we proceeded separately to the research and selection of the studies with the removal of the overlaps, using reference management software such as EndNote 8.0, with the inclusion of the studies. In the third phase, we proceeded to compare the included studies and to resolve any conflicts between the 4 reviewers with the help, if necessary, of a 5th and 6th reviewer (G.T. and A.B.) to decide on doubtful situations. The keywords used were miR-196, miR-196 AND HNSCC, microRNA AND HNSCC, LSCC AND miR-196, OSCC AND miR-196, OPSCC AND miR-196, and HSCC AND miR-196.

The search was performed on 4 different databases: Science Direct, PubMed database, and Cochrane; the gray literature in Open Gray and Google Scholar (keywords miR-196) were also consulted for sources not otherwise identifiable, and systematic reviews on the miR-196 were investigated in search of further records.

Specifically, below are all the keywords used on PubMed: (“miR-196”[All Fields] AND (“hnsccs”[All Fields] OR “squamous cell carcinoma of head and neck”[MeSH Terms] OR (“squamous”[All Fields] AND “cell”[All Fields] AND “carcinoma”[All Fields] AND “head”[All Fields] AND “neck”[All Fields]) OR “squamous cell carcinoma of head and neck”[All Fields] OR “hnscc”[All Fields])) OR “miR-196”[All Fields] OR “MicroRNA-196”[All Fields] OR (“LSCC”[All Fields] AND “miR-196”[All Fields]) OR ((“mouth neoplasms”[MeSH Terms] OR (“mouth”[All Fields] AND “neoplasms”[All Fields]) OR “mouth neoplasms”[All Fields] OR (“oral”[All Fields] AND “cancer”[All Fields]) OR “oral cancer”[All Fields]) AND “miR-196”[All Fields]) OR ((“opscc”[All Fields] OR “opsccs”[All Fields]) AND “miR-196”[All Fields]). Translations HNSCC: “hnsccs”[All Fields] OR “squamous cell carcinoma of head and neck”[MeSH Terms] OR (“squamous”[All Fields] AND “cell”[All Fields] AND “carcinoma”[All Fields] AND “head”[All Fields] AND “neck”[All Fields]) OR “squamous cell carcinoma of head and neck”[All Fields] OR “hnscc”[All Fields]. Oral cancer: “mouth neoplasms”[MeSH Terms] OR (“mouth”[All Fields] AND “neoplasms”[All Fields]) OR “mouth neoplasms”[All Fields] OR (“oral”[All Fields] AND “cancer”[All Fields]) OR “oral cancer”[All Fields]. OPSCC: “opscc”[All Fields] OR “opsccs”[All Fields].

The record search was completed on 12 March 2022, and a final update on the search was performed on 14 May 2022.

### 2.4. Data Collection Process, Data Characteristics

The data to be extracted from the included articles were decided in advance by the four reviewers and concerned the first author of the study, the date of publication, the country where the research was conducted, the type of squamous cell carcinoma, the number of patients, the miRs investigated, the value or type of cut-off between low and high expression for miR-196, clinical characteristics of patients and tumors included in the studies (age, gender, smokers, HPV positive, staging, grading, differentiation, follow up), RR, and the HR values for the different prognostic survival indices.

Moreover, if only the Kaplan–Meier survival curve was present, the HR was calculated using the Tierney method, by extrapolating the data from the curve with Engauge Digitizer 4.1 (open-source, non-commercial project, https://markummitchell.github.io/engauge-digitizer/, accessed on 30 May 2022), and reported in a special Excel spreadsheet available online as Supplemental Materials to the publication of Tierney et al. [32].

### 2.5. Risk of Bias in Individual Studies, Summary Measures, Summary of Results, Risk of Bias between Studies, Additional Measures

The risk of bias in the individual studies was assessed by two authors (M.D. and S.C.), as a tool for the assessment parameters derived from the Reporting Recommendations for Prognostic Studies of Markers (REMARK). The studies with a high risk of bias were considered for exclusion from meta-analysis [33,34].

The evaluation of the heterogeneity was performed through the Higgins index (*I*^2^) and the chi^2^; values of *I*^2^ higher than 75% led to a moderate heterogeneity of the data in the studies. The heterogeneity was also evaluated graphically, across the analysis of the overlapping of the confidence intervals in the forest plot, through the graphical analysis of the funnel plot to search for any sources of heterogeneity on the presence of a publication bias.

The risk of bias between the studies was assessed, graphically through the analysis of the overlaps of the confidence intervals, across the *I*^2^ inconsistency index (an *I*^2^ value greater than 75% was considered high and an analysis of the random effects), and the funnel plots. The possibility of performing a sensitivity analysis was also evaluated in order to identify and exclude the source of heterogeneity to investigate its effects on HR pooled. A subgroup analysis was performed with different meta-analyses data, as a function of the various prognostic indices of survival, and as a function of the histological subtypes of HNSCC.

For the meta-analysis, and in particular for the calculation of the pooled HR, the software Reviewer Manager 5.4 (Cochrane Collaboration, Copenhagen, Denmark) was used. In particular, the GRADE pro-Guideline Development Tool online software (GRADEpro GDT, Evidence Prime, Hamilton, ON, USA) was used to assess the quality of the evidence [35]. The trial sequency analysis (TSA) was performed using Stata 13 (StataCorp, College Station, TX, USA), with the implementation of the R 4.2 software, and by installing the idbounds and metacumbounds commands.

For more support of the scientific evidence on the role of miR-196a and miR-196b as a prognostic biomarker, the TGCA (the Cancer Genome Atlas) database containing a cohort of patients with HNSCC was consulted in order to extract the HR values relating to the prognostic indices linked to the expression of miR-196a and miR-196b [36,37].

## 3. Results

### 3.1. Selection of Studies

**Figure 1:**
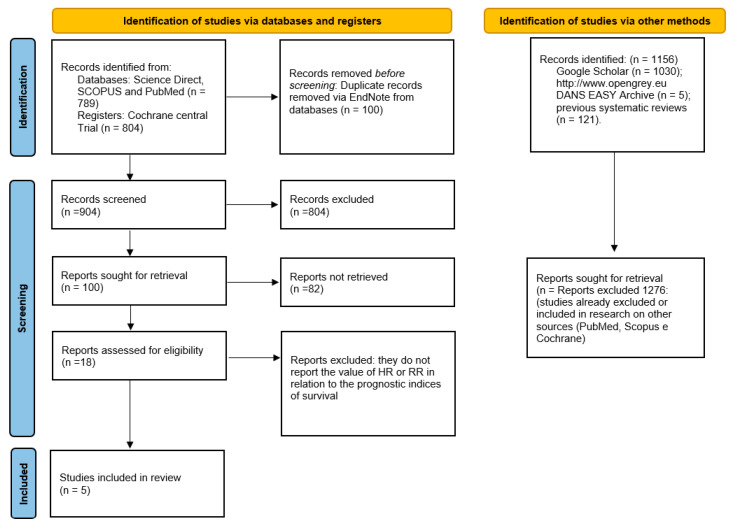
The entire selection and screening procedures are described in the flowchart.

The search in Science Direct, SCOPUS, PubMed, and Cochrane Central trial databases resulted in a number of bibliographic sources equal to 1593. With the removal of duplicates, 904 were obtained, and the articles that were potentially eligible were 18, of which only 5 fully complied with the inclusion and exclusion criteria, and the related extracted data were included in the meta-analysis. Furthermore, the gray literature analysis (http://www.opengrey.eu, accessed on 14 May 2022, DANS EASY Archive and Google Scholar) and previous systematic reviews did not allow to identify additional studies to be included in the meta-analysis (Figure 1). The entire procedure of identification, selection, and inclusion of the studies is indicated in the flow chart of Figure 1.

### 3.2. TGCA Cohort Analysis Results

**Figure 2:**
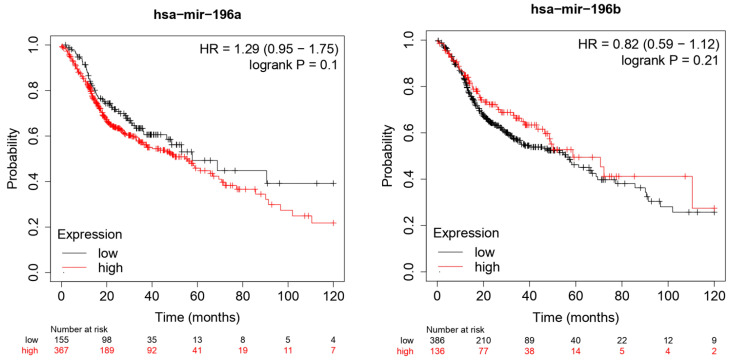
Kaplan–Meier curves according to the miR-196 expression levels for overall survival (OS) in patients with HNSCC (TGCA cohort). Kaplan–Meier curves created by the public database and web application Kaplan–Meier plotter (http://kmplot.com/analysis/, accessed on 2 June 2022).

The TGCA cohort analysis through the Kaplan–Meier Plotter database portal (https://kmplot.com/analysis/, accessed on 2 June 2022), on a cohort of 512 HNSCC patients, generated the following Kaplan–Meier curves (Figure 2) between high and low miR-196 expression. For the cut-off value between high and low expression, the median was selected as a parameter taking into account the follow-up period of 120 months.

The bioinformatic analysis of miR-196a reported an HR for OS between high and low expression of 1.29 (0.95–1.75) log rank *p*-value 0.1, with a median survival for low expression cohort (months) of 57.73, and for high expression cohort (months) of 54.7. On the other hand, the analysis of miR-196b reported an HR for OS 0.82 (0.59–1.12) log rank *p*-value 0.21, with a median survival for low expression cohort of 55.7 and for high expression cohort of 58.73. A limitation to this analysis derives from the fact that the *p*-value of the log rank tests is higher than 0.05 with low statistical significance (Figure 2).

### 3.3. Data Characteristics

At the end of the choice and selection phase, the number of articles that met the eligibility criteria and that presented data that could be included in the meta-analysis was five: Qin et al. [38], Liu et al. [18], Maruyama et al. [39], Zhao et al. [29], and Luo et al. [40].

The data extracted from the articles are reported in Table 1, and the types of data are described in the Section 2.

The total number of patients included in the studies was 417, of which 319 were females. Mean age reported in the articles was approximately 60 years, and the maximum follow-up period was 97 months for the study by Zhao et al. [29]; of a total of 417 HNSCCs, 192 were LSCC, while the rest were OSCC (excluding 3 OPSCC), of which 105 were TSCC.

Zhao et al. [29] and Liu et al. [18] do not report information on the presence of smokers or on the habitual use of alcohol; none of the included studies reported information on HPV positivity. A total of two studies, investigated miR-196b, reporting prognostic survival data (Zhao et al. [26], Luo et al. [35]), and three investigated miR-196a (Qin et al. [33], Liu et al. [19], Maruyama et al. [34]). None of the included studies reported RR data, while the HR data were available in the Maruyama et al. [39] and Luo et al. [40] studies. Following these reports, in the present study, an estimated HR value was calculated starting from the Kaplan–Meier curves, using the method described by Tierney, and the prognostic indices present in the studies were OS (four studies) and DFS (two studies).

### 3.4. Risk of Bias in Studies

The risk of bias of the included studies was evaluated using a classification derived from REMARK [41]. In addition, each parameter was considered as adequate, inadequate, or not evaluable on the basis of the REMARK guidelines. On the basis of the REMARK guidelines, a score from 0 to 3 was considered for each factor (Table 2).

### 3.5. Meta-Analysis

Following the data extracted from the included articles, two meta-analyses were performed in relation to the two main prognostic indices of survival (OS and DFS), and subsequently a subgroup analysis was performed according to the investigated miR (mir-196a, miR-196b). The first meta-analysis concerned OS, and, in particular, HR between high expression and low expression of miR-196 in HNSCC data were extracted from four published articles, and in only two cases the data were estimated from Kaplan–Meier curves (Maruyama et al. [39], Luo et al. [40]).

Heterogeneity for OS was low, with a Higgins index (*I*^2^) equal to 0%, which is why a fixed-effects model was applied.

**Figure 3:**
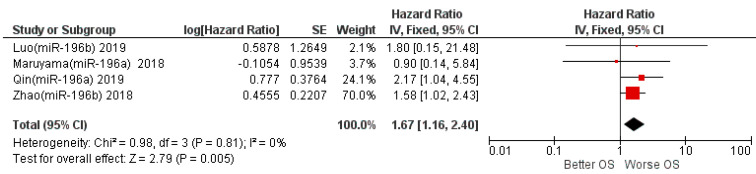
Forest plot of the fixed-effects model of the meta-analysis; OS, HR = 1.67, 95% CI: [1.16, 2.49]; chi^2^ = 0.98 (*p*-value = 0.81); df = degrees of freedom; *I*^2^ = Higgins heterogeneity index, *I*^2^ < 50%, heterogeneity irrelevant; *I*^2^ > 75%, significant heterogeneity; C.I. = confidence intervals; P = *p*-value; SE = standard error. The graph for each study shows the lead author, the date of publication, and the hazard ratio with confidence intervals, with the log HR standard error and weight of each study expressed as a percentage. The final value is expressed in bold with the relative confidence intervals. The black line shows the position of the average value, and the rhombus in light black shows the measure of the average effect. Luo (miR-196b) 2019 [40], Maruyama (miR-196a) 2018 [39], Qin (miR-196a) 2019 [38], Zhao (miR-196b) 2018 [29].

The results of the first meta-analysis reported aggregate HR for OS between high and low miR-196 expression of 1.67, with the relative intervals of confidence [1.16–2.49]; heterogeneity was evaluated through chi^2^ = 0.98, df = 3 (*p* = 0.81), and the Higgins index *I*^2^ = 0%; testing for the overall effect was Z = 2.79 (*p* = 0.005). The forest plot presents the black diamond in a position of worsening OS in relation to the high miR-196 expression (Figure 3).

For the second meta-analysis, which concerned DFS, a random-effects model was applied in light of the high heterogeneity (*I*^2^ = 74%) between the only two studies included (Luo et al. [35], Maruyama et al. [39]).

**Figure 4:**
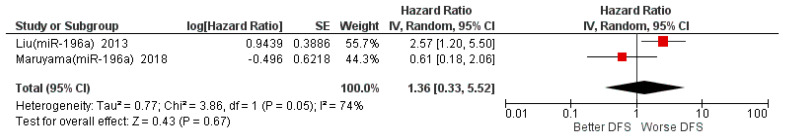
Forest plot of the fixed-effects model of the meta-analysis; DFS, HR = 1.39, 95% CI: [0.33 5.52]; chi^2^ = 3.86 (*p*-value = 0.05). Liu (miR-196a) 2013 [18], Maruyama (miR-196a) 2018 [39].

Aggregate HR for DFS between high and low miR-196 expression was 1.36 with intervals of confidence [0.33; 5.52]; heterogeneity was evaluated through Tau^2^ 0.77, chi^2^ = 3.86, df = 1 (*p* = 0.05), and the Higgins index *I*^2^ = 74%; testing for the overall effect was Z = 0.43 (*p* = 0.67). The final result is that, even if the HR data were in favor of a slight worsening of the DFS, the central rhombus intersects the central line of the non-effect; with the two studies reporting opposite HR results, the data were therefore devoid of statistical significance (*p* = 0.67) (Figure 4).

### 3.6. Risk of Bias across Study, Subgroup Analysis, Publication Bias

**Figure 5:**
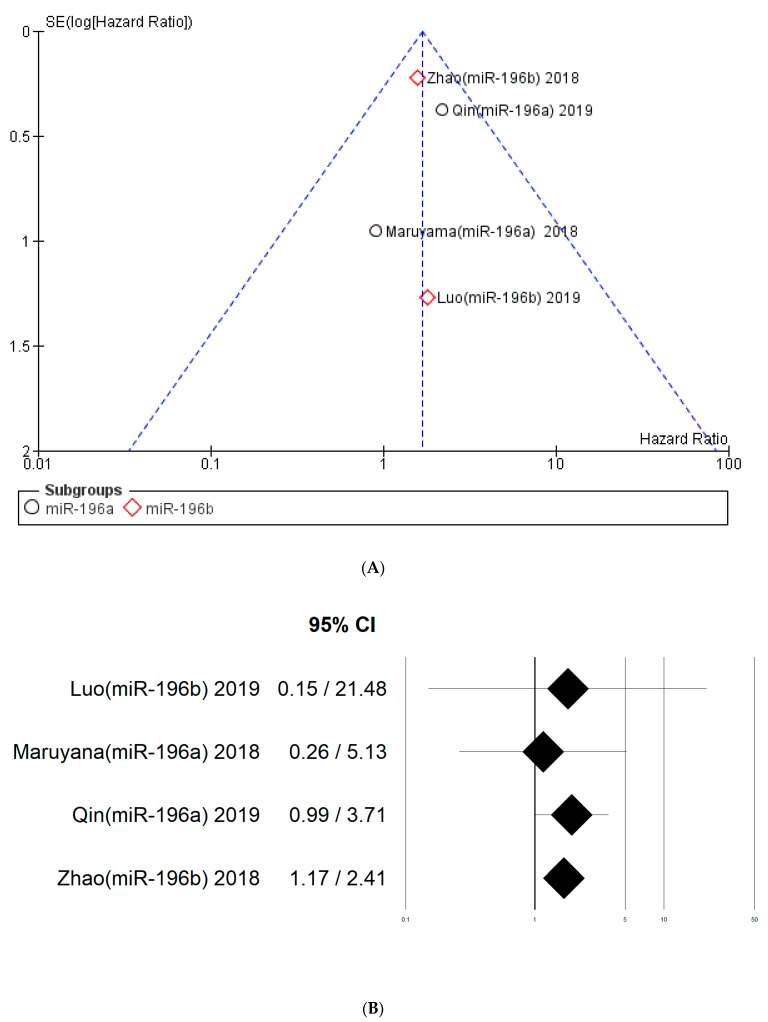
Funnel plot for the first meta-analysis. (**A**) The absence of heterogeneity is highlighted graphically. *I*^2^ = 0%. SE: standard error. (**B**) Confidence interval: 95%; Luo(miR-196b) 2019 [40], Maruyama (miR-196a) 2018 [39], Qin (miR-196a) 2019 [38], Zhao (miR-196b) 2018 [29].

The risk of bias between the studies is considered particularly low. Considering the first meta-analysis, there is also an overlap of the confidence intervals between the various studies; the publication bias is very likely given the small number of included studies. In the visual analysis of the funnel plot there is symmetry in the position of the studies (Figure 5A,B); moreover, all adequate and suitable measures were adopted in order to minimize the failure to find data in the literature, by consulting abstracts of conferences and all unpublished material for lack of significance. For the second meta-analysis, any assessment of the risk of bias is considered superfluous given the inclusion of only two studies.

**Figure 6:**
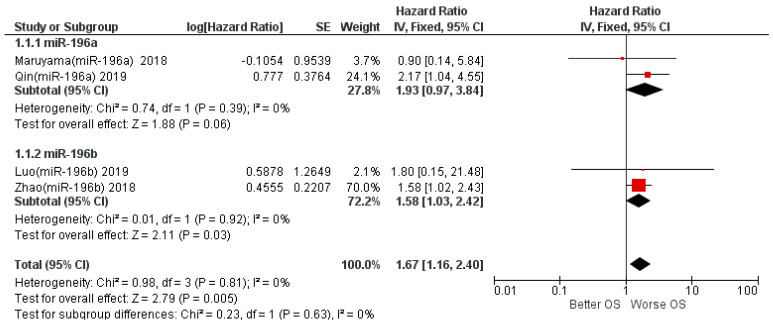
Forest plot of the fixed-effects model of the subgroup meta-analysis for OS, test for subgroup differences. Chi^2^ = 0.23 (*p*-value = 0.63), *I*^2^ = 0%; Luo (miR-196b) 2019 [40], Maruyama (miR-196a) 2018 [39], Qin (miR-196a) 2019 [38], Zhao (miR-196b) 2018 [29].

A subgroup analysis was also conducted by dividing the studies that identified miR-196a and miR-196b. In the first subgroup (miR-196a), the analysis favored a worsening of survival for patients with high expression of miR-96a tissue (HR 1.93), while for the second subgroup, which concerned miR-196b expression data, the data are also slightly favorable to the worsening of OS (HR 1.58), as reported in Figure 6.

The two subgroups, taken individually, were quite homogeneous in *I*^2^ = 0% and *I*^2^ = 0%; the difference between the two subgroups was low, with chi^2^ = 0.23, *I*^2^ = 0%.

A subgroup analysis was not performed according to the histological subtype, since the first subgroup miR-196a was made up only of LSCC, while the second subgroup miR-196b was composed mainly of OSCC. The results would therefore have been comparable to the first analysis of the subgroups: in fact, it emerges that there is no differentiation from these data according to the histological subtype.

### 3.7. Trial Sequential Analysis, Grade

**Figure 7:**
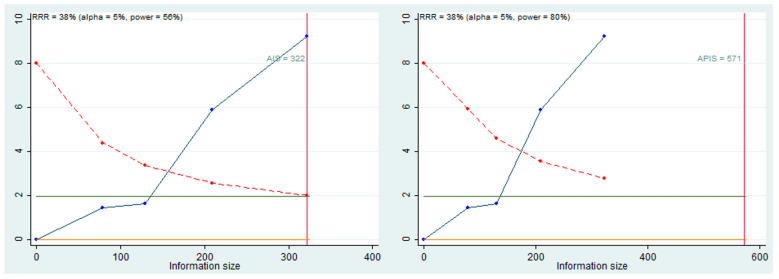
Trial sequential analysis of trials reporting HR between high and low miR-196 expression; AIS, APIS, light green line (Z = 1.98), dashed red line (monitoring boundary, UB), blue line (cumulative z curve). The cumulative z curve was constructed using a random-effects model, red line (sample size). The APIS graph showed that for an RRR of 38%, alpha 5%, and a power of 80%, the number of optimal patients is 571. Crossing of the monitoring boundary before reaching the information size provides firm evidence of effect. This is a true positive result.

Trial sequential analysis (TSA) was performed to evaluate the potency of the result of the meta-analysis (Figure 3), adjusting the results to avoid type I and II errors. The program used was Stata 13 (StataCorp, College Station, TX, USA), with the integration of the R 4.2 software, through the metacumbounds commands, as described by Miladinovic et al. [42]. The O’Brien–Fleming spending function was used by applying random effects (Table 3).The APIS (a priori information size) and, subsequently, the AIS (accrued information size) commands were used via the dialog box to determine the optimal sample size and the power of the results, assuming an RRR (reduction risk relative) of 38%, an alpha value equal to 5% (type 1 error), and beta at 20% (type 2 error) (Figure 7).

The TSA curve crossed the line Z = 1.98, and the crossing of the monitoring boundary before reaching the information size provided firm evidence of effect. The APIS graph showed that for an RRR of 38%, alpha 5%, and a power of 80%, the number of optimal patients is 571.

The authors, through the GRADE Guideline Development Tool (GRADEpro GDT), assessed the quality of the meta-analysis (HR of OS between high and low miR-196 expression) and subgroups result (Table 4).

The results suggested that the quality of the evidence was low.

## 4. Discussion

This systematic literature review was conducted following the PRISMA guidelines, and a meta-analysis and a TSA were also performed on the role of the different expression of miR-196 as a predictive biomarker of survival in HNSCC. The prognostic indices that were the subject of meta-analysis were OS and DFS, of which the HR between high and low expression was calculated; the systematic review included a total of 5 studies with a total number of 417 patients enrolled.

In the literature, there is only one previous systematic review with meta-analysis, on cervical cancer [43], in which tissue expression of miR-196 was investigated as a potential prognostic biomarker (OS). The meta-analysis for miR-196 included only two studies, with the following HR results (HR = 0.28, 95% CI: 0.15–0.52, *p* < 0.001; *I*^2^ = 0%, *p* = 0.950, *n* = 197) [43]. In addition, there were three meta-analyses on miR-196a polymorphism as a risk of developing cancer [44,45,46].

The data analysis of previous and recent systematic reviews revealed how there is the possibility that miR-196a and miR-196b may represent a potential prognostic biomarker [47] in addition to their diagnostic potential. In fact, Cheng et al. (2021), in an bioinformatics data analysis of two databases (TCGA-HNSC dataset and GSE31277 dataset), identified for miR-196b an area under the curve (AUC) of 0.767 [48]. These sensitive and specific data are in agreement with a previous study by Lu et al. (2015) on circulating miR-196a and 196b, with an AUC of 0.963 for oral cancer [49].

For better support of the scientific evidence on the role of miR-196a and miR-196b as a prognostic biomarker, we preliminarily performed a bioinformatic analysis on the TGCA cohort, which counts about 512 samples from patients with HNSCC.

The TGCA cohort analysis reports interesting data for OS, with an HR between low and high miR-196a expression of 1.29 for a follow-up period of 120 months: the high expression would seem to be an unfavorable prognostic index, while for the miR-196b the HR was 0.82; in an opposite way in this case, the high expression of miR-196b seems to be a favorable prognostic index of survival (Figure 2).

The prognostic value of miR-196 tissue expression has been investigated for many neoplasms with conflicting results: in fact, for colorectal cancers the expression of miR-196b is related to poor prognosis and survival [50]. Similar results are also obtained for miR-196a in ovarian neoplasms: indeed, a study conducted by Zhao et al. [51] reported an HR between low and high expression of 0.19 for OS data, in agreement with a study on glioblastoma with an HR between high and low expression of 2.81 [21], while for osteosarcoma the data in the literature would suggest a worsening of survival during downregulation [22].

In the field of head and neck tumors, the data in the scientific literature point towards a worsening of the survival prognosis. In particular, of the five studies included in this systematic review, four are in favor of a worsening of the prognostic indices in the course of high expression, while only one is in favor of a worsening of the prognosis in the case of low expression. In particular, the study in question is the one conducted by Maruyama et al. [39] who, in a cohort of 50 TSCC, found worse survival in both DFS and OS in the group of patients who had low miR-196a expression. However, this study presented data in which TSCC were, at the time of diagnosis, in clinical stage I and II, unlike the other included studies which presented HNSCC with patients also in clinical stages III and IV [34].

Indeed, Liu et al. [18], alternatively for miR-196a, identified a DFS with an HR of 2.57 between high and low expression in a cohort of 95 patients with OSCC, data in agreement with Qin et al., who found for the OS an HR of 2.1 out of 80 OSCC [18].

The data on miR-196b from two studies (Zhao et al. [29], Luo et al. [40]) are also in agreement with those for miR-196a, reporting, respectively, HR values of 1.57 and 1.8 for the OS between high and low expression.

Aggregating the data of the four studies that investigated OS, the HR result is 1.67 in favor of OS worsening, while for the two studies that measured DFS as a prognostic survival index (Maruyama et al. [39] and Liu et al. [18]), the meta-analysis of the data reports an HR result of 1.37, slightly in favor of worsening, but with the confidence intervals that intersect the no-effect line (central rhombus, forest plot in Figure 3). In fact, the two studies are placed towards the final effect in a position of near-equilibrium; however, further evaluations would be superfluous given the low number of studies and patients included in the half-analytic calculation.

The subgroup analysis that separately considered the two miR members of the same non-coding RNA family (miR-196a and miR-196b) reported data in both cases in favor of a worsening of survival, with HR of 1.93 and 1.58, respectively; furthermore, dividing the studies according to the histological subtype OSCC and LSCC, we find the same studies included in the previous subgroup division, with identical HR values.

For the first meta-analysis that considered OS as a prognostic value, it was decided to perform the TSA for the evaluation of the analytical power of the data and, assuming an RR of 38%, the four included studies provide results with adequate statistical power.

The prognostic biomarkers play a potentially fundamental role in guiding us towards an improvement in health in patients diagnosed with carcinoma, and in all those aspects that involve clinical practice, development, and health research, helping the oncologist in identifying the most appropriate possible therapeutic intervention.

The understanding of the prognostic potential of miR-196a and miR-196b could be useful to the clinician after the formulation of the diagnosis to define the prognosis with the creation of prediction models of the individualized prognostic risk. Translating this concept into clinical practice, in a patient with HNSCC who has an unfavorable prognostic molecular signature (with low OS or RFS), a more or less aggressive therapeutic or surgical treatment could be recommended by customizing the therapeutic approach (personalized medicine).

The execution of systematic reviews with meta-analyses data and TSA of Phase 2 prognostic studies can lead to an improvement in the design, execution, and reporting of these studies, and allow us to better highlight the results that are potentially more reliable, trying not to leave out data that are devoid of statistical significance and evidence (publication bias). Consequently, the present systematic review places itself, together with other systematic reviews on this topic, in a key role to the finding of Phase 3 clinical trials studies, in the search for a prognostic model [52].

The limitations of this meta-analysis are identified as the low number of included articles, which only provided five included studies. The correct and scrupulous research methodology that rigorously respected the Cochrane Handbook and the registration of the Research Protocol on PROSPERO, in addition to the analysis of the gray literature, made it possible to identify a greater number of articles compared to previous systematic reviews on the role of miRs as prognostic biomarkers in HNSCC, which included only two studies related to miR-196, along with other non-coding RNAs.

For the other meta-analysis limits, firstly, data were extracted from published results rather than from individual patients’ records. In addition, for the survival outcomes considered in the meta-analysis, a very high rate of heterogeneity was detected that strongly limits the quality of evidence, despite the inclusion of an adequate number of studies performed in a good-quality manner. This heterogeneity could reflect the wide variation of miR-196 expression in the HNSCC population that limits its use as prognostic biomarker in clinical practice: this topic should be evaluated in further studies with different design.

Another limitation of this meta-analysis is that in some studies, the HR value was estimated starting from the Kaplan–Meier survival curves (Table 1), whose methodology is described in the Cochrane Handbook, as reported by Tierney et al. [32].

## 5. Conclusions

In conclusion, with the limitations of the meta-analysis, it can be argued that miRs of the miR-196 family could be independent prognostic biomarkers of survival for HNSCC. Consequently, exhaustive investigations of miRNA, for instance, regarding intercommunication among miRNAs and between miRNAs and other genes, altered protein expression induced by miRNAs, and site-specific miRNA expression profiling, are therefore prerequisite prior to future clinical trials and possible therapeutic applications. However, there is still a long way to go before illuminating their concrete function and mechanisms in HNSCC.

## Figures and Tables

**Table 1:** The data extracted for the four articles included in the meta-analysis.

Lead Author, Data	Country	Study Design	Average Age (M), Years (Y), N (Number of Patients)	Number of Patients Male (M), Female (F)	Grading (G1, G2, G3)	Staging (S) I–II, III–IV	Smoking History (Sm) Yes (Y), No (N)	Alcohol History Al Yes (Y), No (N)	Follow Up Max Months (m) or Range (R)	Tumor Type, Tumor Site	Cut-Off	miR	HR miR-196 Low and High Expression (OS, PFS, CSS, DFS, RFS)
Qin 2019 [38]	China	Prospective	≥60 Y: 39 N <60 Y: 41 N	80 (43 M, 37 F),	\	S I–II: 33 S III–IV: 47	Sm Y: 30 Sm N: 50	Al Y: 24 Al N: 56	80 m	OSCC 80 (Tongue 30 Gingival 24 Cheek 13 Floor of Mouth 10 Oropharynx 3)	median	miR-196a	OS: HR ° 2.175 (1.455–4.034), *p* = 0.039
Liu 2013 [18]	Taiwan	Prospective	M: 53.6 Y	95 (90 M, 5 F)	\	S I–III: 26 S IV: 69	\	\	85 m	OSCC 95 (Buccal mucosa 34, Tongue 25, Others 36)	median	miR-196a, miR-196a2	DFS: HR 2.57 (1.20–5.48), *p* = 0.02
Maruyama 2018 [39]	Japan	Retrospective	<60 Y: 21 N ≥60 Y: 29 N	50 (24 M, 26 F),	G1 ^4^: 31 G2: 16 G3: 0 G4: 1	S I: 32 S II: 18	Sm Y: 19 Sm N: 31	Al Y: 22 Al N: 25	6o m	OSCC (TSCC ^1^) 50	median	miR-196a, miR-10a miR-10b miR-196b	OS HR 0.91 (0.12–7.19) ^2^; DFS: HR 0.6 (0.18–2.06) ^2^
Zhao 2018 [29]	China	Prospective	<60 Y: 42 N ≥60 Y: 71 N	113 (96 M, 17 F),	\	S II: 47 S III–IV: 66	\		R: 40–97 m	LSCC 113 (Glottic 70, Supraglottic 43)	median	miR-196b	OS: HR ^3^ 1.577 (0.989–2.516), *p* = 0.039
Luo 2019 [40]	China	Prospective	M: 60.58 Y	79 (66 M, 13 F),	G1: 11 G2: 32 G3: 36	S I–II: 23 S III–IV: 56	Sm Y: 52 exSm: 21 Sm N: 6	Al Y: 58 exAl: 17 Al N: 4	R: 5–60 m	LSCC 79	median = 4.922	miR-196b	OS: HR 1.80 (0.38–8.51) ^2^

° Multivariate analysis (HR), univariate: OS: HR 3.187 (1.507–6.743) *p* = 0.002; ^1^ tongue squamous cell carcinoma, ^2^ data were extrapolated from the Kaplan–Meier curve according to the Tierney method, ^3^ multivariate analysis (HR), univariate: OS: HR 1.673 (1.098–2.547) *p* = 0.017, ^4^ Maruyama uses the following scale for the histological grade: G1/well, G2/moderate, G3/poor, G4/undifferentiated.

**Table 2:** Assessment of risk of bias within the studies, with scores 8 to 10 = low quality, 11 to 14 = intermediate quality, and 15 to 18 = high quality.

Lead Author, Data	Sample	Clinical Data	Marker Quantification	Prognostication	Statistics	Classical Prognostic Factors	Score
Qin 2019 [38]	2	3	2	2	3	2	14
Liu 2013 [18]	2	1	2	2	3	2	12
Maruyama 2018 [39]	2	2	2	3	2	3	14
Zhao 2018 [29]	3	1	2	2	3	2	13
Luo 2019 [40]	2	3	3	2	2	2	14

**Table 3:** Cumulative random-effects meta-analysis of 4 studies with Lan–DeMets bounds (UB: upper boundary, Z: cumulative Z score, partN: patient. *p* val = *p*-value). The Z-value is the test statistic and |Z| = 1.96 corresponds to *p* = 0.05.

Trial	Estimate	Z	*p* Val	PartN	UB
Luo (miR-196b), 2019 [40]	6.050	1.423	0.155	79	4.376
Maruyama (miR-196a), 2018 [39]	3.408	1.610	0.107	129	3.356
Qin (miR-196a), 2019 [38]	7.306	5.894	0.000	209	2.558
Zhao (miR-196b), 2018 [29]	5.476	9.206	0.000	322	1.990

**Table 4:** Evaluation of GRADEpro GDT; CI, confidence interval; HR, hazard ratio.

Certainty Assessment							No. of Patients	Effect		Certainty
No. of Studies	Study Design	Risk of Bias	Inconsistency	Indirectness	Imprecision	Other Considerations	miR-196	Relative (95% CI)	Absolute (95% CI)	
miR 196										
4	Randomized trials	not serious	not serious	not serious	Serious ^2^	publication bias strongly suspected ^1^	-/322	HR 1.67 (1.16 to 2.40)	2 fewer per 1000 (from 2 fewer to 1 fewer)	⨁⨁◯◯Low
miR subgroups—miR-196a										
2	Randomized trials	not serious	not serious	not serious	Serious ^2^	publication bias strongly suspected ^1^	-/130	HR 1.93 (0.97 to 3.84)	2 fewer per 1000 (from 4 fewer to 1 fewer)	⨁⨁◯◯Low
miR subgroups—miR-196b										
2	Randomized trials	not serious	not serious	not serious	Serious ^2^	publication bias strongly suspected ^1^	-/192	HR 1.58 (1.03 to 2.42)	2 fewer per 1000 (from 2 fewer to 1 fewer)	⨁⨁◯◯Low

^1^ The low number of included studies preponderates to a possible publication bias; ^2^ in 2 studies the hazard ratio was estimated starting from the Kaplan–Meier survival curves, which are not error-free.

## Data Availability

Not applicable.

## Supplementary Materials

The following supporting information can be downloaded at: https://www.mdpi.com/article/10.3390/life12081269/s1, File S1: target genes for miR-196a and miR-196b.
